# Phosphorylated c-Src in the nucleus is associated with improved patient outcome in ER-positive breast cancer

**DOI:** 10.1038/sj.bjc.6604768

**Published:** 2008-11-18

**Authors:** E J Campbell, E McDuff, O Tatarov, S Tovey, V Brunton, T G Cooke, J Edwards

**Affiliations:** 1 Division of Cancer Sciences and Molecular Pathology, Department of Surgery, Glasgow Royal Infirmary, Glasgow, UK; 2 Department of Pathology, Western Infirmary, Glasgow, UK; 3 Cancer Research UK, Beatson Laboratories, Garscube Estate, Glasgow, UK

**Keywords:** ER-positive breast cancer, c-Src activation, nuclear, tamoxifen

## Abstract

Elevated c-Src protein expression has been shown in breast cancer and *in vitro* evidence suggests a role in endocrine resistance. To investigate whether c-Src is involved in endocrine resistance, we examined the expression of both total and activated c-Src in human breast cancer specimens from a cohort of oestrogen receptor (ER)-positive tamoxifen-treated breast cancer patients. Tissue microarray technology was employed to analyse 262 tumour specimens taken before tamoxifen treatment. Immunohistochemistry using total c-Src and activated c-Src antibodies was performed. Kaplan–Meier survival curves were constructed and log-rank test were performed. High level of nuclear activated Src was significantly associated with improved overall survival (*P*=0.047) and lower recurrence rates on tamoxifen (*P*=0.02). Improved patient outcome was only seen with activated Src in the nucleus. Nuclear activated Src expression was significantly associated with node-negative disease and a lower NPI (*P*<0.05). On subgroup analysis, only ER-positive/progesterone receptor (PgR)-positive tumours were associated with improved survival (*P*=0.004). This shows that c-Src activity is increased in breast cancer and that activated Src within the nucleus of ER-positive tumours predicts an improved outcome. In ER/PgR-positive disease, activated Src kinase does not appear to be involved in *de novo* endocrine resistance. Further study is required in ER-negative breast cancer as this may represent a cohort in which it is associated with poor outcome.

Adjuvant hormone therapy results in substantial improvements in disease free and overall survival for woman with early hormone receptor-positive breast cancer (Early Breast Cancer Trialists’ Collaborative Group, 1998, 2005). Despite these benefits, a substantial proportion of patients will develop *de novo* or acquired resistance to hormone therapy and this presents a significant clinical problem.

The precise molecular mechanisms involved in breast cancer cell resistance to endocrine therapy is poorly understood but there is strong evidence suggesting it involves cross talk between the oestrogen receptor (ER), growth factor receptors and other downstream cellular signalling pathways (Osborne and Schiff, 2004) resulting in ligand-independent activation of the ER and tumour cell growth. Indeed, we had earlier shown that HER 1–3 expression is significantly associated with early relapse in an ER+, tamoxifen-treated cohort (Tovey *et al*, 2005). Evidence is now emerging that endocrine resistance not only results in oestrogen-independent growth but is also associated with altered cell–cell and cell–matrix adhesive interactions, promoting a more invasive phenotype (Hiscox *et al*, 2006).

c-Src non-receptor tyrosine kinase is overexpressed and activated in a large number of human malignancies and the relationship between activation and cancer progression appears significant (Irby and Yeatman, 2000). The precise mechanism of its action has not been fully elucidated, but c-Src is known to interact with a diverse array of molecules, including growth factor receptors and cell–cell adhesion receptors, integrins and steroid receptors including the ER (Ishizawar and Parsons, 2004; Shupnik, 2004) promoting tumour cell proliferation, survival, differentiation, migration and invasion (Frame, 2004; Yeatman, 2004). Recent *in vitro* studies have shown the overexpression and over activity of Src during the acquisition of tamoxifen resistance in ER-positive cell lines (Hiscox *et al*, 2005; Planas-Silva *et al*, 2006). Src inhibition was seen to significantly reduce the invasive behaviour of these cells. In addition, inhibition of c-Src has been shown to reduce the incidence of breast cancer metastases and increase survival in mice. Progress in knowledge of c-Src in tumour genesis has resulted in Src kinase inhibition being investigated as a therapeutic target for anti-invasive therapies in breast cancer (Rucci *et al*, 2006; Finn *et al*, 2007).

This study, using a large cohort of ER-positive (+) tamoxifen-treated patients, was undertaken to examine if c-Src expression is involved in *de novo* resistance to tamoxifen treatment. We examine the role of c-Src expression in human ER-positive breast cancers, to determine if *in vivo* c-Src expression, activation or cellular location is associated with response to tamoxifen therapy and patient survival.

## Material and methods

### Patients and tissues

The local ethics committee granted ethical approval for this study to utilise a database that details the outcome of ER-positive patients diagnosed with primary operable breast cancer between 1980 and 1999 treated with adjuvant tamoxifen. Within this cohort all patients received adjuvant tamoxifen (mean time 4.8 years), 26% of patients received adjuvant chemotherapy and 18% received adjuvant radiotherapy. Formalin-fixed paraffin-embedded tissue, taken at the time of surgical resection, was used for tissue microarray (TMA) construction, as described earlier (Tovey *et al*, 2005).

### Immunohistochemistry

**Figure 1:**
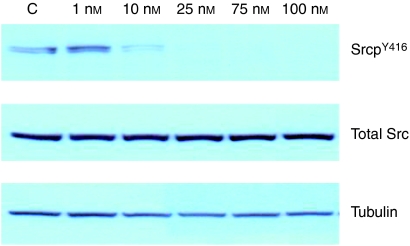
Western blot experiment. Phospho-specific antibody recognising activated c-Src (SrcpY^416^) is shown as a single 60 kDa band (lane 1: control, C). In addition, phosphorylation of c-Src was observed to decrease after treatment with increasing concentrations of the Src kinase inhibitor dasatinib (lanes2–6) and total Src was not affected by this. Tubulin was used as a control.

Immunohistochemistry was performed on 10 normal breast sections and 10 prostate cancer samples, in addition to the 262 ER-positive breast cancer specimens. Full activation of c-Src requires phosphorylation at tyrosine (Tyr) 419 in addition to the absence of phosphorylation at tyrosine 519. A phospho-specific antibody (Cell Signalling Technology, Inc., Danvers, MA, USA) raised in rabbit to phosphorylated Y416, SrcpY^416^, which corresponds to human Tyr 419, was used, as described in the literature (Planas-Silva *et al*, 2006). In addition, an antibody recognising Total Src (36D10, Cell Signalling Technology) was used. Before performing immunohistochemistry, antibody specificity was confirmed by western blotting (Figure 1). As expected, activated c-Src, SrcpY^416^, was detected as a single 60 kDa band and decreased in response to the Src kinase inhibitor dasatinib. Titration of the optimal antibody dilution was undertaken in breast tumour specimens before the procedure.

Tissue sections were dewaxed and rehydrated through graded alcohols and then subject to heat-induced antigen retrieval by pressure steaming in preheated 10 mM citrate buffer for 5 min. Immunostaining was then performed; sections were first treated with hydrogen peroxide and then blocked using horse serum, followed by incubation in primary antibody (1 : 50 dilution, SrcpY^416^ overnight) (1 : 200 for Src36D10, 1 h). DakoCytomation EnVision was applied for 30 min and sections incubated with DAB (1 : 50 dilution). Finally, sections were counterstained, dehydrated and mounted. Positive and negative (isotype-matched antibody) control slides were incorporated in each run.

Tissue-staining intensity was scored blind by two independent observers using a weighted histoscore method (Kirkegaard *et al*, 2006) also known as the Hscore system (McCarty *et al*, 1986). Histoscores were calculated from the sum of (1 × % cells staining weakly positive)+(2 × % cell staining moderately positive)+(3 × % cells staining strongly positive) with a maximum of 300. Each cellular location was separately assessed with a weighted histoscore assigned to any membrane, cytoplasm and nucleus staining. The histoscores for each core were then averaged. Where one core was missing the remaining core(s) scores were used. To determine high and low expression the median value for all scores was used. The inter-class correlation coefficient (ICCC) for each protein was calculated to confirm consistency between observers.

### Western blot analysis

MCF-7 cells treated with varying concentrations of dasatinib were lysed in RIPA buffer (50 mM Tris pH7.6, 150 mM sodium chloride, 1% Triton X-100, 0.5% deoxycholate, 0.1% SDS, 10 mM sodium fluoride, 1 mM sodium orthovanadate and 1 : 100 Calbiochem protease inhibitor cocktail set 1) and centrifuged at 12 000 rpm for 10 min, the supernatant was removed and protein concentration determined using BCA/CuSO_4_ assay. A total of 40 *μ*g of protein per well was resolved by 4–12% gradient Bis-Tris gel electrophoresis (Invitrogen, Paisley, UK); proteins were transferred to nitrocellulose membranes (Millipore, Watford, UK), which were blocked for 1 h in 5% BSA and probed with primary antibodies: anti-phospho SrcY^419^ (1 : 10 000) and anti-total Src (1 : 10 000 Cell Signalling Technologies, UK) at 4°C overnight. Membranes were then incubated with secondary antibodies (anti-rabbit 1 : 5000 or anti-mouse 1 : 5000, Cell Signalling Technologies) and visualised with ECL kit (Amersham, Bucks, UK). Where necessary, the membranes were stripped by incubating with Re-Blot Plus stripping buffer (Chemicon, Hampshire, UK) before re-probing with other antibodies including anti-*β-*tubulin (HRA) (1 : 8000, Abcam, Cambridge, UK) to confirm equal protein loading.

### Statistical analysis

The statistical software package SPSS version 11.5 was used for all analysis. Interclass correlation coefficient was employed to confirm consistency between observers. Protein expression data were not normally distributed and are shown as median and inter quartile ranges. Pearson's χ^2^ test was employed to assess association between staining intensity and known clinical parameters, and survival analyses were conducted using Kaplan–Meier method, curves were compared with the log-rank test. Hazard ratios (HR) were calculated using Cox regression analysis.

## Results

### Clinical and pathological characteristics

Clinical and pathological characteristics for all patients (*n*=262), including age, grade, nodal status, histology, size and Nottingham Prognostic Index are detailed in Table 1. The mean duration of tamoxifen therapy was 4.82 years. Altogether, 55 patients (21%) had breast cancer-specific deaths, 77 patients (29.4%) had breast cancer relapse and 60 of these patients were receiving tamoxifen therapy.

### Localisation of Total Src and activated c-Src in normal breast

Ten normal breast sections were stained for total Src and activated c-Src. Low expression of total Src was observed in the cytoplasm of 60% and nucleus of 40%, however no activated c-Src expression was observed at any location.

### Localisation of activated c-Src expression in ER-positive breast cancer tissue

**Figure 2:**
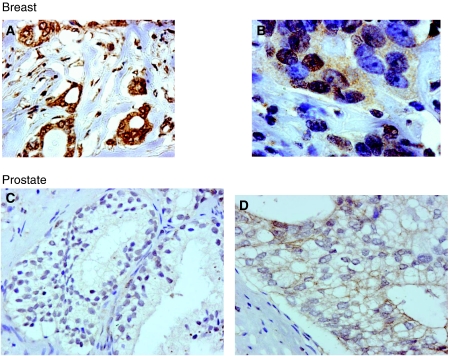
The different localisation of activated c-Src, SrcpY^416^ in prostate and breast tumour samples are shown. In breast tumours, activated c-Src was most commonly present in the cell cytoplasm and the cell nucleus (**A** and **B**). In the prostate cancer (**C** and **D**), the majority of staining observed for phosphorylated c-Src was located to the membrane, and nuclear expression was rarely observed.

A total of 262 ER-positive tumour samples were analysed for activated c-Src expression. 57.3% (150 out of 262) of tumours expressed activated Src in the cytoplasm; median histoscore 20 (interquartile range 0–61.5). 58.4% (153 out of 262) of tumours expressed activated c-Src in the nucleus; median histoscore 10 (interquartile range 0–45). High levels (greater than the median value) of activated c-Src expression in the cytoplasm or nucleus was therefore detectable in over 50% (*n*=153) of all ER-positive breast tumours analysed. 2.7% (7 out of 260, two samples missing) of tumours expressed activated Src in the membrane, median histoscore 0. Because of the low rate of membrane expression observed, it was not deemed appropriate to apply these results to further statistical tests. To confirm that the antibody was able to detect membrane staining 10 prostate tumours were also stained for activated c-Src. Activated c-Src was much more commonly located to the cell membrane of prostate cells compared with the ER-positive breast carcinomas. Figure 2 illustrates the staining patterns observed in the ER-positive breast cancer specimens compared with prostate cancer specimens.

### Activated c-Src and patient outcome

**Figure 3:**
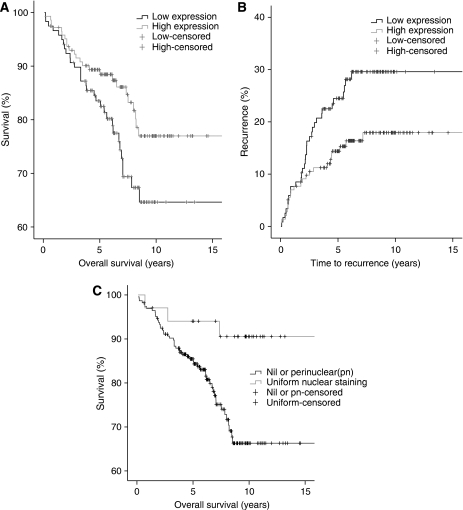
Kaplan–Meier survival curves show (**A**) overall survival difference between ER+ patients with high (above the median value) and low expression of activated nuclear c-Src (*P*=0.047); (**B**) 1−survival curve showing disease recurrence in ER+ patients with high and low expression of activated nuclear c-Src (*P*=0.02) and (**C**) overall survival differences between activated c-Src depending on the pattern of nuclear staining. Uniform nuclear staining was significantly associated with improved survival compared with no nuclear staining or only perinuclear (*P*=0.0153).

High expression level (above the median value) of activated c-Src within the nucleus of tumour cells was significantly associated with improved overall survival (*P*=0.047) and decreased recurrence in tamoxifen-treated patients (*P*=0.02), Figure 3A and B. On Cox regression analysis this was not shown to be independent of survival or recurrence. The location of activated c-Src around the nucleus was also significant, tumours with uniform staining had improved outcome in comparison with patients with only perinuclear staining (Figure 3C, *P*=0.0153). Activated c-Src within the cell cytoplasm was not significantly associated with patient outcome.

### Activated c-Src and prognostic indices

Activated c-Src within the nucleus was associated with node negativity and low NPI (Pearson's χ^2^, *P*=0.03 and *P*=0.046, respectively). Activated c-Src within the cytoplasm of cells was not associated with nodal status, NPI, tumour grade or size. No significant correlation was found with Ki67 (proliferative index). In contrast when the cohort was subdivided by progesterone receptor (PgR) status (histoscore >10), activated c-Src nuclear expression remained highly significant in the ER and PgR+subgroup (*n*=165, *P*=0.004). However, in the ER+/PgR-negative subgroup significance was lost (*n*=93, *P*=0.56). Progesterone receptor status was not available for four tumours from our cohort of 262 patients. The cohort was not stratified for HER2 status as only four tumours were found to be positive using the Herceptest.

### Total Src expression in ER-positive breast cancer

Of the 262 patients only 231 tumour samples were scored for total Src expression. 95.8% (220 out of 231) of tumours expressed total Src in the cytoplasm, median histoscore 97 (interquartile range 40–150). 70.6% (153 out of 230) of tumours expressed total Src in the cell membrane, median histoscore 26 (interquartile range 0–95). No total Src was seen within the cell nucleus. Total Src expression (at any location) was not significantly associated with any clinical parameters or patient outcome.

## Discussion

Although cell line studies strongly support the role of c-Src in endocrine-resistant breast cancer progression, translational studies investigating human breast tumour expression, activation and correlation with clinical parameters are surprisingly limited. Using a large cohort of ER-positive breast cancer patients treated with adjuvant tamoxifen we have shown that high levels of activated c-Src are present in over 50% of tumour specimens and we also show that nuclear c-src activation is significantly associated with improved overall and disease-free survival. Subgroup analysis shows that this benefit is only seen in ER+/PgR+ patients and not within ER+/PgR-negative group.

As c-Src is a non-receptor tyrosine kinase that is localised to the intracellular membranes and cytoplasm of the cell, (Biscardi *et al*, 2000) it was surprising that in this study we rarely observed activated c-Src in the cell membrane. However, antibody specificity was confirmed by western blotting. A single 60 kDa band suggested that phosphorylated Src kinase was detected. In addition, phosphorylation of c-Src (but not total c-Src) was observed to decrease after treatment with increasing concentrations of the Src kinase inhibitor dasatinib confirming that the antibody detected only phosphorylated Src (Figure 1). Although these experiments confirmed that the antibody used in the study was specific for phosphorylated Src kinase, it did not answer the question about the location of phosphorylated Src observed in this cohort. We therefore stained prostate tumours to assess the localisation of activated Src in a different tumour type. In prostate cancer, the majority of staining observed for phosphorylated Src was located to the membrane and nuclear expression was rarely observed. These results suggest that the lack of membrane staining and high level of nuclear staining observed in this study was associated with our ER-positive breast cancer cohort, and was not a characteristic of the antibody used. However, the Y^416^ sequence is highly conserved among the src kinases so this does not exclude detection of other src family kinases along with c-Src using this antibody. Our detection of nuclear c-src expression and activation is in line with recent literature as c-Src has been reported both within the nucleus and nucleolus (David-Pfeuty *et al*, 1993; David-Pfeuty and Nouvian-Dooghe, 1995) of other solid tumours. Earlier immunohistochemical work showed that in non-malignant breast cells c-Src is distributed within the cytoplasm, whereas in malignant breast cells the majority of c-Src appears concentrated around the nucleus (Verbeek *et al*, 1996).

In this study we found that high levels of activated c-Src was present in over 50% tumour specimens analysed and nuclear activated c-Src was significantly associated with improved overall survival and decreased recurrence. Ito *et al* (2002) also found that elevated activated c-Src was inversely correlated with biological aggressiveness in 73 breast cancer specimens and suggested that c-Src may have an important role in malignant transformation of breast cells rather than malignant progression. Madan *et al* (2006) subsequently showed that c-Src activation did not correlate with the development of invasive tumour properties but correlated with malignant transformation. In ductal carcinoma *in situ,* activated c-Src was found to correlate with high tumour grade, high proliferation and HER 2 positivity, suggesting that high c-Src activity may identify a subset of DCIS at risk of disease progression to invasive carcinoma (Wilson *et al*, 2006).

The body of evidence does, however, still support a role for c-Src in malignant progression. Compared with adjacent normal tissues, elevated Src expression and/or activity has been reported in a wide range of tumour types, including breast cancer (Verbeek *et al*, 1996) and in many of these tissues, an increase in Src activity correlates with disease stage or malignant potential (Aligayer *et al*, 2002; Weiner *et al*, 2003). Tumour cell lines possessing elevated Src activity are often highly metastatic, displaying an increased capacity for migration and invasion *in vitro* (Mao *et al*, 1997; Jackson *et al*, 2000; Slack *et al*, 2001).

Recent *in vitro* breast cell line work, show overexpression and over activity of Src during the acquisition of tamoxifen resistance in ER+ cell line (Hiscox *et al*, 2005; Planas-Silva *et al*, 2006). Src inhibition was seen to significantly reduce the invasive behaviour of cells. Hiscox *et al* found that elevated Src kinase activity in endocrine-resistance models was independent of Src gene or protein level. Tamoxifen resistance may be either *de novo* (present before tamoxifen treatment) or ‘acquired’. In this study all analysis was performed on tumour samples taken before tamoxifen treatment, and although we do not find that active c-Src correlates with *de novo* endocrine resistance, it is interesting that within our cohort the survival benefit was only in ER+/PgR+ patients and not in the ER+/PgR negative group. PgR expression is a marker of a functional ER and a number of laboratory studies have shown the importance of molecular characteristics such as PgR and HER2 in predicting tumour response to endocrine therapy. We have reported earlier that ER+/PgR-negative tumours are more likely to relapse on tamoxifen (Tovey *et al*, 2005), and a number of other laboratory studies report a reduction in PgR expression in ER+ cells that is consistent with acquired tamoxifen resistance (Scott *et al*, 2007). It is therefore possible that in tumours with a functioning ER (ER+/PgR+) ‘active’ c-Src is in the nucleus and not able to perform its role in promoting tumour progression. Tumours acquiring tamoxifen resistance over time have an adaptive change in growth factor signalling (such as a reduction in PgR expression, increased EGFR expression), therefore Src kinase being downstream of such signalling networks may not become fully active until later during the development of tamoxifen resistance. High levels of activated c-Src expression in the cell cytoplasm have been reported in recurrent breast carcinoma samples (Planas-Silva *et al*, 2006) although this expression was not compared with the primary tumour sample. Comparison of primary breast tumour c-Src expression with expression in recurrent or metastatic tumours following endocrine resistance would be a preferable model. Within our laboratory we have examined this in prostate cancer specimens. In hormone-sensitive prostate cancer, active c-Src was associated with improved survival but after development of hormone therapy resistance, active c-Src was associated with reduced time to death (unpublished data).

It is also likely that our patient cohort represents a good prognostic group and that the aggressive phenotype associated with Src kinase is limited to poor prognostic cancers. Indeed, Finn *et al* (2007) recently reported a highly significant relationship between breast cancer cell line subtype based on gene expression of cytokeratins and sensitivity to src kinase inhibition, suggesting that the ‘triple negative’ breast cancers were most likely to benefit from Src inhibition. ER-negative tumours correlate with poor tumour differentiation, high proliferation rate and other unfavourable characteristics, and are, in general, considered a more aggressive breast carcinoma. An inverse correlation between Src and ER levels has been reported, ER-negative primary breast cancer and cell lines showed increased Src levels and/or activity compared with ER+cancers (Chu *et al*, 2007).

In conclusion, we found that elevated levels of activated c-Src within the cell nucleus of ER+ breast cancer were associated with improved patient outcome in a large cohort of Tamoxifen-treated ER-positive patients. Although we are unable to substantiate the *in vitro* studies suggesting a role for c-Src in tamoxifen resistance we feel that further clarification defining the role of c-Src in the different subtypes of breast cancer, particularly in ER-negative breast cancer and recurrent tumours, is warranted as this likely represents the group in which targeted Src Kinase inhibition may be beneficial to patient outcome.

## Figures and Tables

**Table 1:** Patient clinical and pathological variables

	**Number**	**Valid%**
*Grade*		
1	60	23.3
2	124	48.2
3	73	28.4
Unknown	5	
		
*Nodal status*		
0	128	53.3
1–3	72	30
4+	40	16.7
Unknown	22	
		
*Histological type*		
Ductal	218	83.2
Lobular	20	7.6
Other (incl. unknown)	24	9.2
		
*Size (mm)*		
T1(<20)	89	36.3
T2 (20–50)	149	59.1
T3 (>50)	14	5.6
Unknown	10	
		
*NPI*		
<3.5	66	35.5
3.5–5.5	94	50.5
>5.5	26	14
Unknown	76	
		
*Age (years)*		
</=50	42	16
>50	220	84
		
*Chemotherapy*		
Yes	68/262	26
No	194/262	74
		
*Progesterone receptor (PgR) status*		
PgR+	165	63
PgR−	93	35
PgR unknown	4	1.5

NPI=Nottingham Prognostic Index (grade+nodal status+0.02 × size in mm).

Grade=Bloom and Richardson grade. Nodal status=number of positive nodes, Histological type: ductal, invasive ductal carcinoma; lobular, invasive lobular carcinoma; other includes mucinous, mucoid etc.
